# DNA structural features of eukaryotic TATA‐containing and TATA‐less promoters

**DOI:** 10.1002/2211-5463.12166

**Published:** 2017-02-16

**Authors:** Venkata Rajesh Yella, Manju Bansal

**Affiliations:** ^1^ Molecular Biophysics UnitIndian Institute of ScienceBangaloreIndia; ^2^ Present address: Department of BiotechnologyK L University, VaddeswaramGuntur522502India

**Keywords:** DNA structural features, G‐quadruplex motifs, promoter, TATA‐box

## Abstract

Eukaryotic genes can be broadly classified as TATA‐containing and TATA‐less based on the presence of TATA box in their promoters. Experiments on both classes of genes have revealed a disparity in the regulation of gene expression and cellular functions between the two classes. In this study, we report characteristic differences in promoter sequences and associated structural properties of the two categories of genes in six different eukaryotes. We have analyzed three structural features, DNA duplex stability, bendability, and curvature along with the distribution of A‐tracts, G‐quadruplex motifs, and CpG islands. The structural feature analyses reveal that while the two classes of gene promoters are distinctly different from each other, the properties are also distinguishable across the six organisms.

**Abbreviations**
AFE: average free energy
TLS: translation start site
TSS: transcription start site

Gene expression is the most fundamental biological process, in which the genetic information is used to create a phenotype. The initiation of transcription is the first and most crucial step in the regulation of gene expression. Promoters are the genomic sequences where the transcriptional machinery assembles, and the core promoter activity is conferred by the presence of short sequence motifs at specific positions relative to the transcription start site (TSS). TATA box, Inr (Initiator), BRE (TFIIB recognition element), DPE (downstream promoter element), MTE (motif ten element), TCT (polypyrimidine initiator), and Sp1 (specificity protein 1) are well‐characterized sequence motifs reported in several eukaryotes (reviewed in 1, 2, 3, 4, 5, 6, 7, 8). The majority of core promoter motifs serve as binding sites for components of the basal transcription machinery, in particular, TFIID and TFIIB 1. Furthermore, few noncanonical promoter elements such as ‘CpG islands’ 9 and ‘ATG deserts’ 10 have also been well characterized in mammals and are more prominent than the canonical promoter elements 4. Although a variety of core promoter architectures have been revealed, the precise biochemical mechanisms that govern transcription initiation events from the constituent elements are still being elucidated 4. TATA box is the best‐characterized core promoter element and is considered as being most ancient since it is present in organisms ranging from yeast to plants and metazoans. The TATA box is usually located at the −30 or −31 position relative to the TSS in metazoans 1, 6 and at −120 to −40 region relative to TSS 2 or −200 to −50 relative to ‘ATG’ start codon in yeast 11. A wide variation is found in the percentage of TATA box‐containing promoters reported in several studies, which is due to differences in the definition used for TATA box, the window size considered for extracting TATA‐containing promoters, and to a lesser extent the datasets used. Analyses of human promoter sequences report 2.0–2.6% 12, 24% 13, and 27% 14 of sequences as TATA box‐containing promoters.

In eukaryotes, genes can be broadly classified as TATA‐containing and TATA‐less based on the presence or absence of a TATA box in their promoter sequences 11. They have been studied in depth in yeast, and it is reported that TATA‐containing genes are expressed at extremely high or low levels (indicating high plasticity), are stress‐induced, and are under evolutionary selective pressure, when compared to TATA‐less genes 11. The two classes of genes also vary in their usage of transcription factors (SAGA vs. TFIID) in yeast 15. Furthermore, in yeast, TATA‐containing genes have a preference for subtelomeric location in the genome and have more duplicates 11, 16. Promoter sequences of the two classes of genes in yeast and flies have also been shown to differ in their nucleosome occupancy, with TATA‐less genes displaying canonical nucleosome occupancy, with nucleosome‐free regions in the immediate upstream of TSS (core promoter region), while the TATA‐containing promoter regions are occupied by nucleosomes 17, 18, 19. In mammals, the two classes adopt different strategies for transcription initiation (focused in TATA‐containing and dispersed in TATA‐less) 5, 20.

Differences in core promoter nucleotide composition and basic gene features such as length of gene, mRNA, and introns in the two classes of genes have been reported earlier 13, 21. However, primary sequence inspection alone provides limited information about the promoter activity. Since, the initiation of transcription involves not only orchestration of different factors but also the DNA–protein recognition, formation of stable complexes and finally the open complex formation, studying DNA structure gives more insights about promoter function. Several studies have shown that promoter regions of both prokaryotic and eukaryotic genomes have distinct structural features compared to their neighboring regions, as well as coding regions 22, 23, 24, 25, 26, 27, 28, 29. Recently it has been reported that the DNA structural features of promoter sequences are linked to gene expression variability in *Saccharomyces cerevisiae* 30. Furthermore, the local intrinsic structural features like groove shape, flexibility, and topography are shown to be more informative than the simple nucleotide sequence in understanding the DNA‐binding specificities of transcription factors 31, 32. Compared to a simple nucleotide sequence, structural features have more information content, as similar sequences can have similar structural properties in a majority of the cases, as well as very different structures in few cases while divergent sequences sometimes can adopt equivalent local structure 31. Along with the unique structural features, promoter regions of human have been shown known to possess an unusually high presence of G‐quadruplex‐promoting sequences 33.

An earlier study has reported differences in DNA bendability of TATA‐containing and TATA‐less promoters in 11 yeast species based on the translation start site (TLS) data of yeast that was available at that time 34. Prevalence of various DNA structural features in the two classes of promoters in different domains of life has not been compared till date. The current study aims to analyze three distinct structural properties, DNA duplex stability, bendability, and curvature in TATA‐containing and TATA‐less promoters in six eukaryotic systems, *S. cerevisiae*,* Caenorhabditis elegans*,* Drosophila melanogaster,* zebrafish, mouse, and human. Along with the structural features, the hexamer composition and occurrence of structural motifs (A‐tracts and G‐quadruplexes) have been analyzed. The similarities and differences in structural features of the two classes of promoters are discussed in this report.

## Datasets and methods

### Promoter sequence sets

Promoter regions of six eukaryotes *S. cerevisiae*,* C. elegans*,* D. melanogaster*, zebrafish, mouse, and human relative to TSS information were obtained from different sources. TSS information of *S. cerevisiae*,* C. elegans*, and *D. melanogaster* (4912, 18 457 and 12 897) was retrieved from Xu *et al*., 2009 35, modENCODE (http://www.modencode.org/) and Graveley *et al*., 2011 36 transcriptome profiling studies, respectively. TSS information of vertebrates, zebrafish, mouse, and human (5366, 16 955, and 29 456, respectively) was obtained from DBTSS database version 7.0 37. Five hundred upstream and downstream sequences relative to TSS have been extracted by mapping to their respective genomes and are referred to as promoter sequences. Whole genome sequences of *S. cerevisiae*,* C. elegans*,* D. melanogaster*, and vertebrates (zebrafish, mouse, and human) are downloaded from SGD (http://www.yeastgenome.org/), Wormbase (https://www.wormbase.org/), Flybase (http://flybase.org/), and UCSC genome browser (https://genome.ucsc.edu/), respectively.

### Extraction of TATA‐containing and TATA‐less promoters in six eukaryotes

Based on several criteria such as a maximum sequence length (8 bp), minimal consensus sequence, confined upstream location, and conservation across orthologous upstream regions, TATA box was defined as TATA(A/T)A(A/T)(A/G) (TATAWAWR) 11. TATA‐containing promoters in *S. cerevisiae* have been defined as those which contain the TATA consensus sequence TATAWAWR in the upstream location −200 to −50 relative to the TLS. In the present study, we have considered TATA‐containing promoters as those sequences which contain TATA box within −150 to −1 region relative to TSS. Table 1 shows the number of promoter sequences for both classes of genes in *S. cerevisiae*,* C. elegans*,* D. melanogaster*, zebrafish, mouse, and human.

**Table 1:** TATA‐containing and TATA‐less promoters in six eukaryotes

	TATA‐containing			TATA‐less		
	Number of Promoters (%)	GC %		Number of Promoters (%)	GC %	
		[−500 to +500]	[−500 to −1]		[−500 to +500]	[−500 to −1]
*S. cerevisiae*	842 (17.1)	39.5	38.7	4070 (82.9)	38.3	37.4
*C. elegans*	1611 (8.7)	35.8	33.5	16846 (91.3)	35.7	34
*D. melanogaster*	1851 (14.4)	42.0	36.9	11046 (85.6)	43.1	39.4
Zebrafish	530 (9.9)	37.4	34.6	4836 (90.1)	38.4	36.3
Mouse	489 (2.9)	48.6	46.7	16466 (97.1)	55.2	53.7
Human	907 (3.1)	44.6	43.2	28549 (96.9)	53.7	52.8

TSS information for *S. cerevisiae*,* C. elegans*,* D. melanogaster*, zebrafish, mouse, and human are obtained from different sources (Datasets and methods). TATA‐containing promoters in the six systems are defined as those which contain the consensus motif TATAWAWR in the −150 to −1 region, relative to the TSS. Percent of TATA‐containing and TATA‐less promoters in the six systems are indicated in parentheses. GC percentages of the −500 to +500 and −500 to −1 regions relative to TSS for two classes of promoters are also given.

### DNA structural feature calculations

The three DNA structural features, DNA duplex stability, bendability (two models; DNase 1 sensitivity and nucleosome positioning preference), and intrinsic curvature have been chosen, as they are biologically relevant, and the information content of each feature is different. The stability of a double‐stranded DNA molecule can be expressed as the sum of free energy or average free energy (AFE) of its constituent base paired dinucleotides. The dinucleotide energy values corresponding to the 16 dinucleotide steps (or 10 unique dinucleotides) are taken from the unified parameters obtained from melting studies on 108 oligonucleotides 38. The protein‐induced bendability of a given sequence has been calculated by using experimentally derived bendability models. Two different trinucleotide models based on DNase 1 sensitivity 39 and nucleosome positioning preference 40 have been used to estimate bendability. Intrinsic static curvature has been computed using in‐house software NUCRADGEN 41 using wedge angles derived from gel retardation studies (BMHT parameters) 42. Structural properties have been calculated using one nucleotide sliding window and converting each promoter sequence into overlapping di/trinucleotide feature values. Window sizes of 15, 30, and 75 have been used for calculating stability, bendability, and curvature, respectively 26, 29. To obtain the structural profiles, all promoter sequences in each class were aligned, relative to their TSSs and then sequence information was converted to numerical values. The numerical values obtained for all sequences were averaged at each nucleotide position, to get the mean structural property for each system.

### A‐tract, G‐tract, G4‐motif, and CpG island calculations

A‐tracts consist of stretches of minimum four consecutive A : T base pairs without a TA dinucleotide step. A‐tracts of length more than five can act as antinucleosomal sequences 43. In this study, A‐tracts of length seven (A7 or T7) were searched in the promoter regions. A G‐quadruplex is a four‐stranded DNA structure with stacked guanine tetrads at its core 44. G‐quadruplex‐forming sequences are predicted from primary DNA sequence. Putative G‐quadruplex motifs were computed using a simple pattern match G_3−5_N_1−7_G_3−5_N_1−7_G_3−5_N_1−7_G_3−5_ or C_3−5_N_1−7_C_3−5_N_1−7_C_3−5_N_1−7_C_3−5_ 45, where *N* indicates the loop regions and can have any nucleotide. CpG islands (CGIs) are short interspersed DNA chunks that deviate significantly from the average genomic pattern by being GC‐rich, CpG‐rich, and predominantly nonmethylated. CpG islands were calculated using Takai and Jones, 2002 algorithm 9, and were defined as regions longer than 500 bp in size, with a GC composition ≥ 55%, and an observed/expected CpG ratio of ≥ 0.65.

## Results and Discussion

Six eukaryotic genomes, *S. cerevisiae*,* C. elegans*,* D. melanogaster*, zebrafish, mouse along with human are considered for this analysis as they are good representative model systems for understanding aspects of eukaryotic transcription at different levels. These six systems differ in their genomic GC content and nucleotide composition, are well studied, and their experimentally validated TSS data have been published. The promoter sequences of six eukaryotes are classified as TATA‐containing and TATA‐less promoters based on the presence or absence of TATA box in the −150 to −1 promoter region relative to TSS (as described in Datasets and methods). The TATA‐containing core promoters constitute ~ 17% of the total promoters in *S. cerevisiae*, ~ 9% in *C. elegans*, ~ 14% in *D. melanogaster*, ~ 10% in zebrafish, and ~ 3% in human and mouse (Table 1). The percentage of TATA‐containing promoter sequences varies from 46% in *S. cerevisiae* to ~ 14% in human if the −500 to +500 region spanning TSS is considered 24. The GC content of the −500 to +500 region in the two systems is found to increase from 38.5% to 53.4% 24. A comparison of the GC content in the −500 to −1 region in TATA‐containing and TATA‐less promoters reveals that it is considerably lower in TATA‐containing promoters when compared to that in TATA‐less promoters, in case of mouse and human, while the difference is much smaller for other systems (Table 1). However, in *S. cerevisiae*, the GC content of this −500 to −1 upstream region in TATA‐less promoters is found to be slightly lower than in TATA‐containing promoters, due to a large number of TATA‐less promoters containing the TATA box elements outside the core promoter region. The structural and compositional features of TATA‐containing and TATA‐less promoters of the six eukaryotes, *S. cerevisiae*,* C. elegans*,* D. melanogaster*, zebrafish, mouse, and human have been analyzed to understand similarities and differences between them.

### TATA‐containing and TATA‐less promoters have distinct structural properties

The average structural properties, DNA duplex stability (or average free energy), bendability (using two models; DNase 1 sensitivity and nucleosome positioning preference) and curvature of TATA‐containing and TATA‐less promoter sequences of yeast, invertebrate and mammals are computed as reported in earlier studies 26, 29.

**Figure 1:**
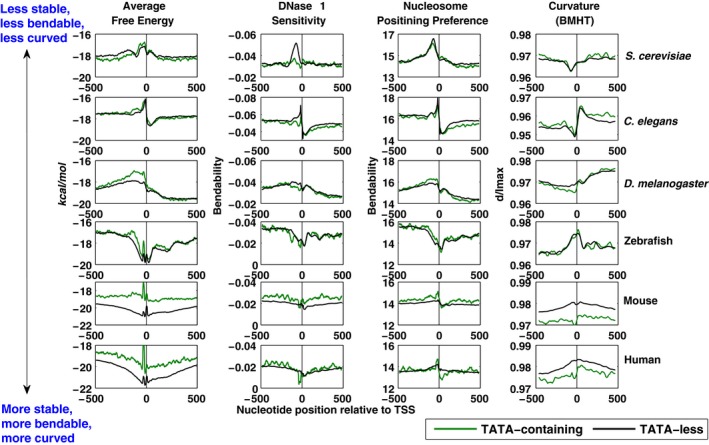
Sequence‐dependent structural properties of TATA‐containing and TATA‐less promoters of six eukaryotes. DNA duplex stability, DNase 1 sensitivity, Nucleosome positioning preference and curvature in promoter regions of *S. cerevisiae*,* C. elegans*,* D. melanogaster*, zebrafish, mouse, and human have been plotted. Structural profiles of TATA‐containing promoter regions are shown in green color while those for TATA‐less promoters are in black. The Y‐axes of DNase 1 sensitivity profiles are reversed for comparison with nucleosome positioning preference model. DNA duplex stability and intrinsic curvature show a significant difference in the two classes of promoters in all six systems.

The average stability profiles of TATA‐containing promoters are different from TATA‐less promoter regions in all six eukaryotes (Fig. 1). The TATA‐containing promoters are less stable compared to TATA‐less promoters, but the span of the low stability region varies in each eukaryote. In *S. cerevisiae*,* C. elegans*, and zebrafish, the TATA‐containing promoters show low stability regions at approximately −150 to −1 region relative to TSS compared to TATA‐less promoters. In mouse and human, a significant difference in the two classes of promoters is observed across the whole region. The TATA‐containing promoters are less stable in entire −500 to +500 region with two sharp peaks at −30 and −1 region. The stability of DNA is directly dependent on AT/GC content. The lower stability of TATA‐containing promoters in mammals is due to their lower GC content as compared to TATA‐less promoters (Table 1). Interestingly, the upstream region (−500 to −150) in TATA‐containing promoters in *S. cerevisiae* shows greater stability, and this correlates with its higher GC content in −500 to −1 region when compared to the TATA‐less promoters, as discussed above and shown in Table 1. Although there is a variation in the shapes of the AFE profiles, the two classes of promoter region show low stability regions or peaks irrespective of the genomes. In order to read the genetic information in DNA by many processes such as replication, repair, recombination, and transcription, the DNA has to be brought transiently into a single‐stranded form. The presence of the low stability in promoter regions in two classes of gene promoters in eukaryotes shows the significance of DNA meltability in genome transcription.

The bendability and curvature profiles also show differences between TATA‐containing and TATA‐less promoters. The bendability profiles of the TATA‐containing and TATA‐less promoters have been analyzed using two models, DNase 1 sensitivity and nucleosome positioning preference model as seen in Fig. 1. The DNase 1 sensitivity model shows that the TATA‐containing promoters are more flexible (or more bendable) compared to TATA‐less promoters in the core promoter regions in *S. cerevisiae*,* C. elegans*,* D. melanogaster*, and zebrafish (Fig. 1), while Satchwell's nucleosome positioning preference model shows that TATA‐containing promoter regions in *D. melanogaster,* mouse, and human to be more rigid. The rigidity of TATA‐less promoters (estimated by DNase 1 sensitivity model) at ~ 100–200 bp upstream of the start codon in 11 yeast species has been reported earlier 34. Our results with TSS data in *S. cerevisiae* and other invertebrates are consistent with this earlier result. DNA bendability describes the anisotropic bending of duplex DNA in the presence of various binding factors. Bendability or flexibility can have two completely different roles. More flexibility is important for several DNA‐binding proteins such as the TATA‐binding protein 46, the catabolite gene activator protein (CAP) 47 and integration host factor (IHF) 48 which play role in transcription and genome organization and the dinucleotide steps TA and CA/TG are frequent in the sequences with increased flexibility 49, 50. Rigid DNA in promoter sequences can play several roles *in vivo*. Rigid or less bendable regions disfavors formation of nucleoids in prokaryotes and nucleosomes in eukaryotes, making these regions ‘nucleosome depleted’ and assist in the assembly of the transcriptional machinery. The rigidity of DNA in promoter regions provides greater scope for sliding of DNA‐binding proteins along its length 34. Furthermore, the higher energy cost required for DNA bending may play a role in open complex formation during transcription initiation, by making the DNA resistant to bending and aiding easy escape of the transcription machinery from promoter region 24.

The average intrinsic curvature profiles of TATA‐containing and TATA‐less promoter regions of *S. cerevisiae*,* C. elegans*, and *D. melanogaster* show that both classes of promoter sequences are curved in the vicinity of TSS with TATA‐containing promoters being slightly more curved (Fig. 1). TATA‐containing promoters in *D. melanogaster* are more curved compared to TATA‐less promoters. The TATA‐containing and TATA‐less promoters in mouse and human have a distinct difference with TATA‐containing promoters being more curved throughout the −500 to +500 region. The biological role of intrinsic curvature of DNA was established in the kinetoplast DNA of trypanosomes 51, and they can enhance transcription rate in bacteria 52, 53. The importance of curvature was recognized in promoter regions of pathogenic bacteria and thermo‐sensing bacteria 54, 55, but it is less apparent in higher eukaryotic promoters.

Of the four structural features studied, stability, and curvature show the most significant differences in the two classes of promoters in mouse and human. In order to get a quantitative estimate of differences in structural properties of TATA‐containing and TATA‐less promoters in core promoter region, −150 to −1 relative to TSS, in all six eukaryotes, cumulative distribution function plots were examined (Fig. S1). These plots, as well as corresponding *P*‐values (shown in Table 2), suggest that the four structural features, average free energy, bendability, and curvature are significantly different in the core promoter regions of all six systems, except for curvature in *S. cerevisiae*. The differences in the structural properties of these two classes of promoters may be attributed to differences in their nucleotide composition as well as sequence, and hence these were examined further.

**Table 2:** TATA‐containing and TATA‐less promoters are distinctly different in their structural properties

	Average free energy	DNase 1 sensitivity	Nucleosome positioning preference	Curvature
*S. cerevisiae*	9.8373 × 10^−14^	1.1455 × 10^−30^	1.1788 × 10^−05^	9.5 × 10^−03^
*C. elegans*	1.2755 × 10^−26^	1.7171 × 10^−47^	1.0664 × 10^−06^	1.8608 × 10^−11^
*D. melanogaster*	6.7109 × 10^−62^	1.8661 × 10^−54^	1.0513 × 10^−57^	3.0452 × 10^−66^
Zebrafish	4.2503 × 10^−11^	1.0092 × 10^−09^	9.4065 × 10^−09^	1.0664 × 10^−06^
Mouse	1.7325 × 10^−64^	1.9847 × 10^−06^	1.8379 × 10^−18^	5.0682 × 10^−68^
Human	5.0682 × 10^−68^	2.8143 × 10^−45^	5.0682 × 10^−68^	5.0682 × 10^−68^

Two‐sample Kolmogorov–Smirnov test (KS‐test) is used to check the statistical significance of the difference between various structural features. The null hypothesis is that the two datasets are different from each other in their cumulative distributions of structural properties in −150 to −1 region relative to TSS (shown in Figure S1). The *P*‐values, at a significance level of *P* ≤ 0.001 suggest that all four structural features, average free energy, bendability (calculated using two models, DNase 1 sensitivity, and nucleosome positioning preference) and curvature are significantly different in all six systems, except for curvature in *S. cerevisiae*.

### TATA‐containing promoters have distinct nucleotide composition compared to TATA‐less promoters

**Figure 2:**
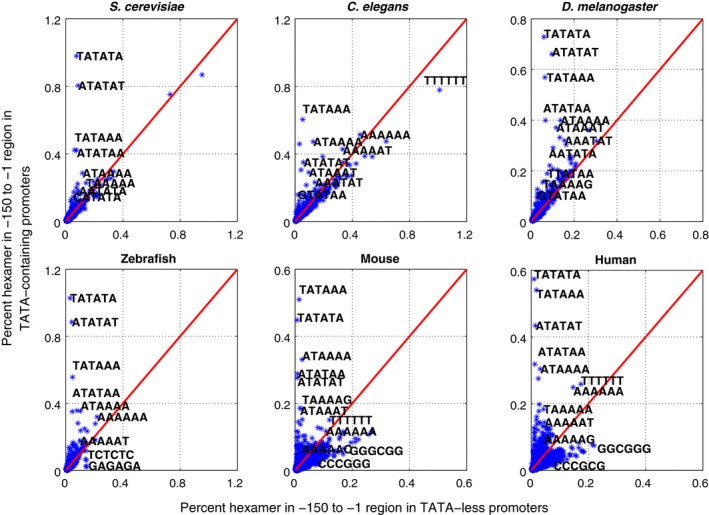
Hexanucleotide composition of TATA‐containing and TATA‐less promoters in six eukaryotic systems. The figure shows the distribution of all possible hexamers in core promoter regions (−150 to −1 w.r.t TSS) of two classes of promoters in *S. cerevisiae*,* C. elegans*,* D. melanogaster*, zebrafish, mouse, and human. Points above the diagonal line correspond to hexamers that are more represented in TATA‐containing promoter regions as compared to TATA‐less promoters. The highly over‐represented hexanucleotides (> 3σ deviated from the best fit line) in each category are labeled.

The characteristic differences in the structural properties of TATA‐containing and TATA‐less promoter regions in six eukaryotes can arise due to the differential base composition and prevalence of some selected oligonucleotides. Hexanucleotide composition has been calculated in the −150 to −1 core promoter region as well as −500 to −1 regions for the two classes of promoters. The unique hexamer words in promoter regions of TATA‐containing and TATA‐less promoter are calculated and compared (Figs 2 and S2).

The TATA‐containing promoters show biases for TA containing hexamer steps (TATATA, ATATAT, ATATAA, and TATAAA) in the −150 to −1 region (Fig. 2 as well as proximal regions (Fig. S2) in all six systems. As expected, the −150 to −1 regions show a high prevalence of TATA motif containing hexamers in all systems, while there is no common motif observed in TATA‐less promoters. The core promoter regions of TATA‐less class in *C. elegans* show preponderance of TTTTTT, whereas mammals show preference for GGGCGG, CGCCCC, CGGGGC, CCCCGC, GGCGGC, and CGCCGC. Zebrafish core promoters in TATA‐less class show a different trend, with a high frequency of hexamers CTCTCT and GAGAGA. The −500 to −1 regions in the TATA‐containing class in mammals have preference for AAAAAA (as well as TTTTTT in human) while GC‐rich hexamers (SP1 elements) are overrepresented in TATA‐less promoters (Fig. S2).

The hexamer distribution in the two classes of promoters is different and quite distinct in the six eukaryotes, even in the −500 to −1 region (Fig. 2). The results suggest that the TATA box‐containing promoters differ in the composition of sequence motifs over a broader region that extends beyond the TATA box‐containing core, especially in mammals. Furthermore, we have studied the frequency of occurrence of different structural motifs A‐tracts, G‐tracts, and G‐quadruplexes along with CpG islands in the two classes of promoter sequences.

### TATA‐containing and TATA‐less promoters have different structural motifs

Table 3 shows the distribution of oligo‐A‐tracts, G‐quadruplex‐forming sequences, and CpG islands in the two classes of promoter regions of six eukaryotic organisms. The percentages of three promoter regions, spanning from −500 to +500, −150 to −1, and −50 to −1, with at least one occurrence of A‐tracts (A7 or T7) and G‐quadruplex‐favoring sequences (G_3−5_N_1−7_G_3−5_ N_1−7_G_3−5_N_1−7_G_3−5_ or C_3−5_N_1−7_C_3−5_N1_−7_C_3−5_N_1−7_C_3−5_) have been calculated. CpG islands have been computed using a 500nt window, so their occurrence is only given for the −500 to +500 region.

**Table 3:** Structural motifs in promoters regions of TATA‐containing and TATA‐less genes of six eukaryotic systems

		*S. cerevisiae*	*C. elegans*	*D. melanogaster*	Zebrafish	Mouse	Human
A‐tracts							
TATA‐containing	−500 to +500	75.3	81.9	54.9	64.7	40.5	55.8
	−150 to −1	40.5	33.7	19.0	16.2	7.6	13.7
	−50 to −1	10.3	12.7	6.1	4.5	2.2	4.1
TATA‐less	−500 to +500	75.5	86.8	53.1	63.2	32.6	38.3
	−150 to −1	40.5	37.8	15.3	13.3	4.8	7.2
	−50 to −1	8.5	16.9	5.0	2.9	1.2	2.4
G‐quadruplex motifs							
TATA‐containing	−500 to +500	0.5	1.1	1.9	1.9	26.8	19.3
	−150 to −1	0.2	0.3	0.2	0.4	7.0	4.4
	−50 to −1	0.1	0.0	0.0	0.0	1.6	0.6
TATA‐less	−500 to +500	0.4	1.6	3.0	1.9	45.2	42.2
	−150 to −1	0.1	0.4	0.4	0.7	16.2	15.1
	−50 to −1	0.0	0.1	0.1	0.1	4.4	4.1
CpG							
TATA‐containing	−500 to +500	0.2	0.3	14	3.6	24.7	18.5
TATA‐less	−500 to +500	0.1	0.2	14.3	5.0	51.3	42.0

Percentage of promoter regions with at least one occurrence of the structural motifs such as A‐tracts (A7 or T7) and G‐quadruplex‐favoring sequences (G_3−5_N_1−7_G_3−5_N_1−7_G_3−5_N_1−7_G_3−5_ or its complementary sequence) have been shown. Three promoter regions, spanning −500 to +500, −150 to −1, and −50 to −1, with respect to TSS (TSS at ‘0’ position) have been considered. CpG islands are calculated using ‘CpG island search’ program and 500nt window 9. TATA‐less promoters in mouse and human are enriched with G‐quadruplex motifs and CpG islands as compared to TATA‐containing promoters.

**Figure 3:**
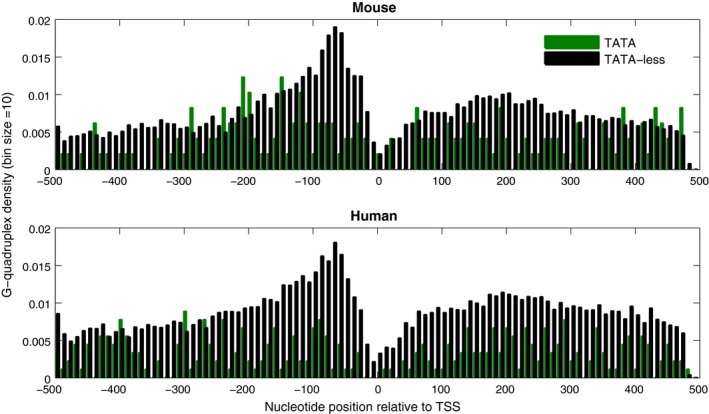
Positional distribution of G‐quadruplex motifs in TATA‐containing and TATA‐less promoter regions of mouse and human. The regular expression G_3−5_N_1−7_G_3−5_ N_1−7_G_3−5_N_1−7_G_3−5_ or C_3−5_ N_1−7_C_3−5_ N1_−7_C_3−5_N_1−7_C_3−5_ is searched in −500 to +500 region relative to TSS and summed for each 10 nucleotide bin. To compare the two classes of promoters, G‐quadruplex motif density has been calculated by dividing the total number of promoters in each bin, which contains a G‐quadruplex motif, by the number of promoter sequences in each class. TATA‐less promoters of mouse and human are enriched with G‐quadruplexes in the vicinity of TSS, as compared to TATA‐containing promoters.

The A7‐tract does not show much difference in distribution in different regions, relative to TSS, for the two classes of promoters in *S. cerevisiae*,* D. melanogaster,* and zebrafish, while in *C. elegans*, A7‐tracts are more prevalent in TATA‐less promoters. In mouse and human TATA‐containing promoters, A‐tracts are comparatively more frequent than in TATA‐less promoters (Table 3). G‐quadruplex motifs are rarely present in both TATA‐containing and TATA‐less promoter regions of *S. cerevisiae*,* C. elegans*,* D. melanogaster*, and zebrafish (Table 3). However, the TATA‐less promoters of mouse and human are significantly enriched in G‐quadruplexes as compared to TATA‐containing promoters (Table 3). Figure 3 shows the positional distribution of G‐quadruplex‐favoring sequences for TATA‐containing and TATA‐less promoters in mouse and human. In mouse and human, approximately ~ 45% and ~ 42% of TATA‐less promoters (in −500 to +500 region relative to TSS) are associated with G‐quadruplexes while only ~ 27% and ~ 19% TATA‐containing promoters contain a G‐quadruplex motif. The G‐quadruplex density in TATA‐less promoters is significantly higher as compared to TATA‐containing promoters in both mouse and human, particularly in the −200 to−1 region (Fig. 3). The G‐quadruplex structures have the potential to influence transcription in both positive (when present on the anti‐sense strand) and negative (when present on sense strand) ways 44, 56, and they could disfavor the assembly into nucleosomal structures 57.

**Figure 4:**
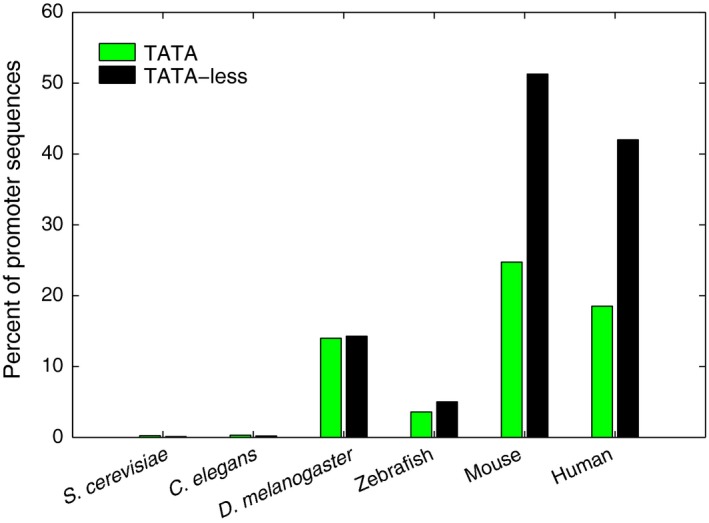
CpG island distribution in TATA‐containing and TATA‐less promoter regions. CpG islands percentage values are calculated using “CpG island searcher” program with 500nt window 9. TATA‐less promoters are enriched in CpG islands as compared to TATA‐containing promoters in mouse and human. In mouse and human approximately ~ 51% and ~ 42% of TATA‐less promoters (in −500 to +500 region relative to TSS) are associated with CpG islands while TATA‐containing promoters have only 25% and 19%, respectively.

The TATA‐containing and TATA‐less promoters also show differences in the distribution of CpG islands which are known to occur frequently in mouse and human. The TATA‐less promoters of mouse and human promoter regions (−500 to +500) are significantly enriched with CpG islands with ~ 51% and ~ 42% containing these elements, while only ~ 25% and ~ 19% of TATA‐containing promoters contain these elements (Fig. 4). CpG islands and high GC content in the mammalian promoter region can favor open chromatin conformation and support paused transcription genome‐wide 58. Furthermore, the GC‐rich sequences can form left‐handed Z‐DNA at alternating purine–pyrimidine stretches 59.

Overall the composition and structural features of TATA‐containing and TATA‐less promoter regions are found to be distinctly different even in regions outside the TATA elements. The differences become more prominent going from *S. cerevisiae* to mammals and may be due to lower mutation rates in TATA‐containing promoters. In the case of mammals, it has been reported that TATA‐containing promoters tend to evolve more slowly in core promoter as well as upstream regions than the promoters that lack a TATA box 5, 60.

## Conclusions

The sequence‐dependent structural properties of di and tri nucleotides in DNA, lead to variations in the structure at a higher level and play a role in protein binding, DNA melting, nucleosome organization, and gene regulation. The structural features of TATA‐containing and TATA‐less promoters are distinctly different in lower eukaryotes. The TATA‐containing core promoters are less stable, more flexible, and more curved compared to TATA‐less promoters in *S. cerevisiae*,* C. elegans*, and *D. melanogaster*. In mouse and human, stability and curvature are distinguishing features of TATA‐containing and TATA‐less promoters. Significant differences are also observed in the distribution of sequence motifs, such as A‐tracts, G‐quadruplexes, and CpG islands, in TATA‐containing and TATA‐less promoters in mouse and human. The TATA‐less promoters in mammals are characterized by high prevalence of G‐quadruplexes and CpG islands. Overall the work reported in this article gives a broad picture of DNA structural and compositional features of two classes of promoters in different eukaryotes and provides interesting insight into their architecture.

## Author contributions

YRV and MB conceived and designed the project. YRV acquired and analyzed the data. YRV and MB interpreted the data and wrote the paper.

## Supporting information


**Figure S1.** Cumulative distribution function of structural features for TATA‐containing and TATA‐less promoters in the six eukaryotic systems: *S. cerevisiae, C. elegans, D. melanogaster,* zebrafish, mouse, and human.

**Figure S2.** Hexanucleotide composition of TATA‐containing and TATA‐less promoter of different eukaryotic systems.
