# Supplementary figures and images for: DNA structural features of eukaryotic TATA‐containing and TATA‐less promoters
Source: FEBS Open Bio. 2017 Feb 16;7(3):324–34. doi: 10.1002/2211-5463.12166 (PMC5337902; doi:10.1002/2211-5463.12166)

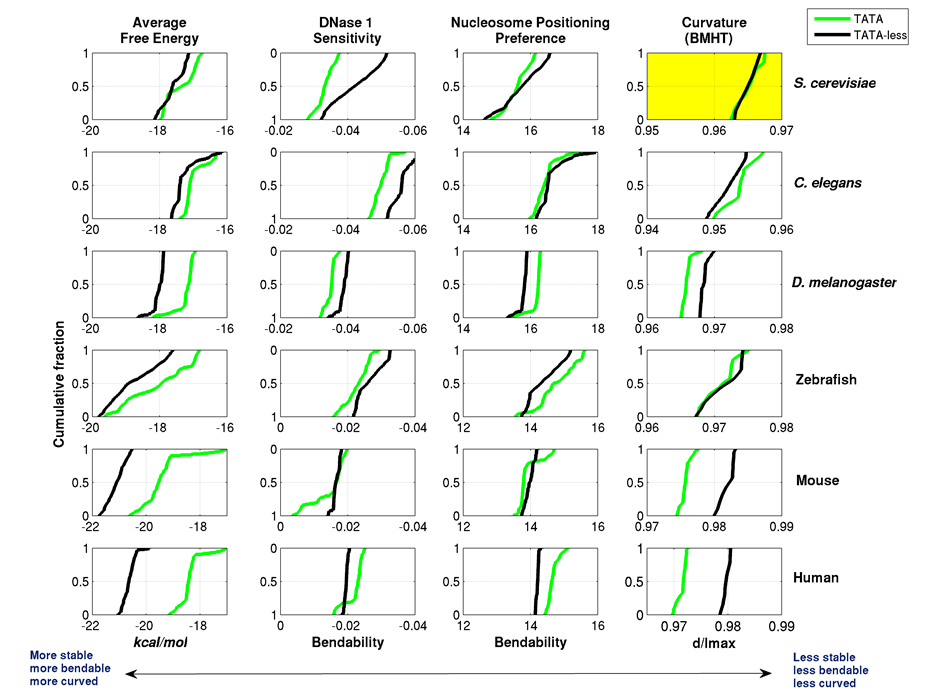

Supplement: Supplementary file 1 — Figure S1. Cumulative distribution function of structural features for TATA‐containing and TATA‐less promoters in the six eukaryotic systems: S. cerevisiae, C. elegans, D. melanogaster, zebrafish, mouse, and human. [file FEB4-7-324-s001.tif]

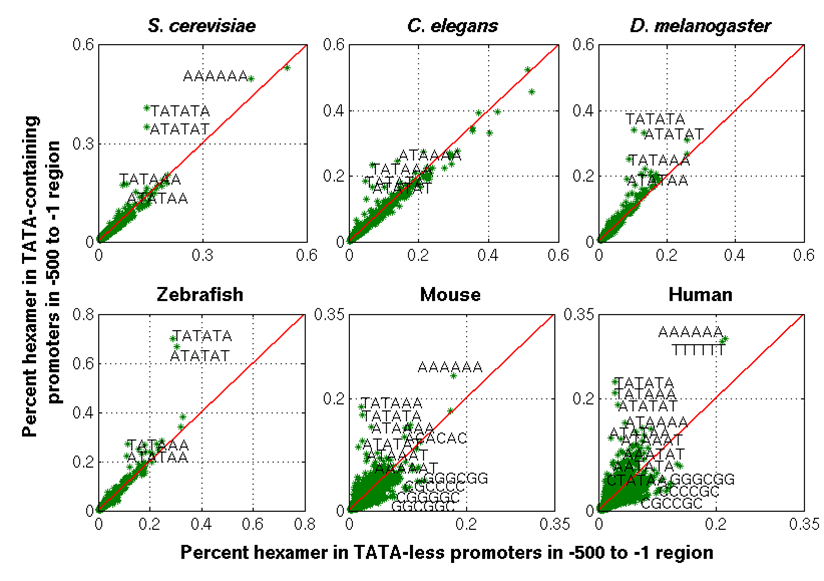

Supplement: Supplementary file 2 — Figure S2. Hexanucleotide composition of TATA‐containing and TATA‐less promoter of different eukaryotic systems. [file FEB4-7-324-s002.tif]
