# Supplementary material for: DNA structural features of eukaryotic TATA‐containing and TATA‐less promoters
Source: FEBS Open Bio. 2017 Feb 16;7(3):324–34. doi: 10.1002/2211-5463.12166 (PMC5337902; doi:10.1002/2211-5463.12166)
Supplement: Supplementary file 3 [file FEB4-7-324-s003.doc]

**Supplementary figure 1**: Cumulative distribution function of structural features for TATA-containing and TATA-less promoters in the six eukaryotic systems: ***S. cerevisiae, C. elegans, D. melanogaster,* zebrafish, mouse, and human.** Empirical cumulative distribution frequency of four structural features for -150 to -1 region relative to TSS are shown for the TATA-containing **(green)** and TATA-less **(black)** promoters. X-axis represents structural property value, while y-axis denotes corresponding cumulative frequency. Plot with yellow background shows the case where the difference in distribution is not significant at p ≤ 0.001 (two-sample Kolmogorov–Smirnov test). The axes of DNase 1 sensitivity profiles have been reversed for comparison with nucleosome positioning preference profiles.

**Supplementary figure 2:** Hexanucleotide composition of TATA-containing and TATA-less promoter of different eukaryotic systems. The figure shows the distribution of all possible hexamers in -500 to -1 region relative to TSS of TATA-containing and TATA-less promoters in S*. cerevisiae, C. elegans, D. melanogaster,* zebrafish, mouse, and human. Points above the diagonal line represent highly represented hexamers in TATA-containing promoter regions as compared to TATA-less promoters. The over-represented hexanucleotides (**>**3σ deviated from the best fit line) in each category are indicated.
